# Feeling coerced during voluntary and involuntary psychiatric hospitalisation: A review and meta-aggregation of qualitative studies

**DOI:** 10.1016/j.heliyon.2023.e13420

**Published:** 2023-02-02

**Authors:** Benedetta Silva, Mizue Bachelard, Joëlle Rosselet Amoussou, Debora Martinez, Charlotte Bonalumi, Charles Bonsack, Philippe Golay, Stéphane Morandi

**Affiliations:** a Community Psychiatry Service, Department of Psychiatry, Lausanne University Hospital and University of Lausanne, Switzerland; b Cantonal Medical Office, General Directorate for Health of Canton of Vaud, Department of Health and Social Action (DSAS), Lausanne, Switzerland; c Psychiatry Library, Education and Research Department, Lausanne University Hospital and University of Lausanne, Switzerland; d General Psychiatry Service, Department of Psychiatry, Lausanne University Hospital and University of Lausanne, Switzerland; e Institute of Psychology, Faculty of Social and Political Sciences, University of Lausanne, Switzerland

**Keywords:** Perceived coercion, Psychiatric hospitalisation, Review, Qualitative study, Meta-aggregation

## Abstract

**Objective:**

This review aimed to provide an aggregative synthesis of the qualitative evidence on patients’ experienced coercion during voluntary and involuntary psychiatric hospitalisation.

**Design:**

A qualitative review.

**Data sources:**

The search was conducted, in five bibliographic databases: Embase.com, Ovid MEDLINE(R) ALL, APA PsycINFO Ovid, Web of Science Core Collection and the Cochrane Database of Systematic Reviews.

**Review methods:**

Following the Joanna Briggs Institute approach, a systematized procedure was applied throughout the review process, from data search to synthesis of results. The reporting of this review was guided by the standards of the PRISMA 2020 statement. The quality of the included studies was critically appraised by two independent reviewers using the JBI Critical Appraisal Checklist. Included findings were synthesized using meta-aggregation. Confidence in the review findings was assessed following the Confidence in the Output of Qualitative research synthesis (ConQual) approach.

**Results:**

A total of 423 studies were identified through the literature search and 26 were included in the meta-aggregation. Totally, 151 findings were extracted and aggregated into 27 categories and 7 synthesized findings. The synthesized findings focused on: the patients' experience of the hospitalisation and the associated feeling of coercion; the factors affecting this feeling, such as the involvement in the decision-making process, the relationships with the staff and the perception of the hospital treatment as effective and safe; the coping strategies adopted to deal with it and the patients’ suggestions for alternatives. All synthesized findings reached an overall confidence score of “moderate”. The seven findings were downgraded one level due to dependability limitations of the included studies.

**Conclusion:**

Based on these findings, seven recommendations for clinical practice where developed, such as fostering care ethics, promoting patients' voice and shared decision-making, and enhancing patients’ perceived closeness, respect and fairness. Five recommendations for future research were also prompted, for instance improving the methodological quality and cultural variation of future qualitative studies, and exploring the psychosocial impact of experienced coercion on patients. For these recommendations to be effectively implemented, a profound change in the structure and culture of the mental health system should be promoted. The involvement of patients in the design, development and scientific evaluation of this change is strongly recommended.

## 1 Introduction

The feeling of being coerced during psychiatric hospitalisation is a profoundly personal and emotionally subjective experience, only partially due to being actually submitted to a formal coercive measure. Indeed, several studies have shown the limit of using formal coercion as a proxy of experienced coercion [[1], [2], [3], [4], [5]].

Inpatients may feel coerced into treatment because of more subtle non-statutory forms of coercion they subjectively experience during the admission process. Many studies have highlighted that professionals frequently resort to the use of treatment pressures [6], also called “informal coercion” [7], in order to promote treatment adherence and avoid formal coercive measures [3,[8], [9], [10]]. Professionals’ unwelcomed predictions, advices, offers and expectations may also be perceived as a threat if expressed in coercive environments and stressful situations [6,[11], [12], [13]]. Moreover, many patients feel compelled to voluntarily accept treatment because of their fear that refusal may result in the use of coercion, also known as the “coercive shadow” [14].

A thorough understanding of experienced coercion and the factors influencing it is essential, especially in light of the strong negative impact it may have on therapeutic relationship [15,16], patients' cooperation [17] and patients’ satisfaction with treatment [[18], [19], [20], [21]], which in turn affects other outcomes, such as engagement with services and adherence to treatment [[22], [23], [24], [25]]. Jordan and McNeil (2019) found that the risk of suicide attempts after discharge increased in patients reporting a higher level of perceived coercion during admission [26].

Given the risk at stake as well as the worldwide increasing use of coercion [27], over the past few years a large number of studies have addressed the issue of perceived coercion and its determinants in order to develop interventions and clinical practices able to reduce it.

The aim of this paper is to provide an aggregative synthesis of the qualitative evidence on patients' experienced coercion during voluntary and involuntary psychiatric hospitalisation based on which to develop better-targeted recommendations for clinical practice and research on how to prevent and reduce perceived coercion. The decision to focus the review on qualitative studies stems from the proven ability of this approach to capture the most specific and in-depth details of such a subjective and complex experience [28]. Previously, other qualitative reviews on similar topics have been published. However, most of them focused only on the experiences of psychiatric patients’ undergoing involuntary treatment and hospitalisation, and did not consider the experience of coercion of voluntarily admitted patients [[29], [30], [31], [32], [33]]. Other reviews addressed specific forms of coercion [[34], [35], [36]] or precise aspects of the admission experience [37]. Finally, some reviews explored the literature on experienced coercion in specific countries [38] or included studies exploring coercion experienced in outpatient settings and by different stakeholders [39]. Because of what mentioned above about the limit of formal coercion as a measure of experienced coercion, a new synthesis including studies on both voluntarily and involuntarily admitted patients is warranted in order to provide a broader understanding of this phenomenon. Moreover, to the best of our knowledge, this is the first review on this topic that uses meta-aggregation to synthesize qualitative evidence [[40], [41], [42]]. Meta-aggregation is an approach to qualitative synthesis that “mirrors the accepted conventions for systematic review whilst holding to the traditions and requirements of qualitative research” [43]; (p. 23). Similarly to meta-analysis, meta-aggregation aggregates the findings of qualitative studies into a whole that tries to be more than the sum of the independent findings. Grounded in the philosophical tradition of pragmatism [41], the main objective of meta-aggregation is not to reinterpret the results of original studies but to summarize them to produce generalizable statements that lead to recommendations for research, practice and policies [40].

## 2 Methods

### 2.1 Study design

In order to explore patients’ feeling of being coerced during hospital admission, we conducted a literature review and synthesis of qualitative studies following the Joanna Briggs Institute approach [43]. The reporting of this review was guided by the standards of the PRISMA 2020 statement [44]. A systematic procedure was applied throughout the review process, from data search to synthesis of results.

### 2.2 Inclusion criteria

#### 2.2.1 Type of participants

This review included all qualitative studies exploring coercion experienced by adult psychiatric patients during admission to a psychiatric hospital. Since the feeling of being coerced can be due to both formal and informal coercion, studies involving involuntarily as well as voluntarily admitted patients were taken into account. Involuntary admission was defined as the legal process through which a person can be detained against their will in a psychiatric hospital. All diagnostic groups were eligible, with the exception of organic disorders and mental retardation. Furthermore, studies were excluded if they focused exclusively on people affected by eating disorders or learning disabilities. Studies on mixed samples, such as patients, professionals and relatives, were considered only if the results for the patients’ subsample were clearly distinguishable.

#### 2.2.2 Phenomena of interest

The current review considered all studies whose main topic was coercion as subjectively experienced and perceived by patients during the hospitalisation process. Thus, papers were excluded if they focused on: 1. other issues of the hospitalisation process; 2. the experience of specific coercive measures, such as seclusion, restraint or forced medication; 3. specific stages of the hospitalisation, such as tribunals’ hearings, assessment or transport under mental health legislation without exploring the broader process.

#### 2.2.3 Context

Only studies exploring the experience of coercion during admission to a psychiatric hospital were eligible. Studies including other settings, such as outpatient services, nursing home, forensic hospitals, or other residential facilities were not considered.

#### 2.2.4 Type of studies

Papers based on qualitative data were included in the review regardless of the study paradigm (interpretive or critical) and design (ethnography, phenomenology, grounded theory, etc.). Descriptive qualitative studies were also eligible. Qualitative components of mixed methods studies were considered if the qualitative findings were reported separately. Papers reporting on the same sample were eligible if the analyses were performed from a new perspective and with different objectives. In these cases, only the new themes were taken into account in the synthesis.

### 2.3 Search strategy

The search strategy was developed with the support of a medical librarian (JRA). The search was conducted on June 15, 2022, in five bibliographic databases: Embase.com, Ovid MEDLINE(R) ALL, APA PsycINFO Ovid, Web Of Science Core Collection and the Cochrane Database of Systematic Reviews. No language or date restrictions were applied. Additional records were identified through backward citation chasing. The detailed search strategies are available in Supplementary file 1.

### 2.4 Study selection

After removal of duplicates, two researchers (B.S. and M.B.) independently screened all titles and abstracts. In case of disagreement, study inclusion was discussed in the presence of a third reviewer, until agreement was reached. Only peer-reviewed articles written in English, French or Italian were assessed. Systematic reviews, books, book chapters, theses and dissertations, reports and commentaries were excluded. Relevant studies were read in full and screened based on the predefined inclusion criteria.

### 2.5 Quality assessment

The methodological quality of the studies considered for inclusion was assessed using the JBI Critical Appraisal Checklist [43]. This instrument evaluates the congruity between the study methodology and the stated philosophical perspective, research question, methods to collect data, representation and analysis of the data, and interpretation of the findings. The degree to which the researcher values, beliefs and influences are made explicit, the adequate representation of the participants' voices, the relationship between these voices and the authors' conclusions and the ethics of the study are also considered [40]. Two independent reviewers critically appraised the quality of the included studies. In case of disagreement, study appraisal was discussed in the presence of a third reviewer, until agreement was reached. No study was excluded based on its quality but only if participants’ voices were not adequately represented in the results (question #8; at least one illustration per theme).

### 2.6 Data extraction and synthesis

Data extraction and synthesis were performed using the NVivo 1.5 software. Following the JBI approach [45], data extraction was performed in two steps. First, the characteristics of each included study, such as author(s), year of publication, country, aim, methods and type of participants, were extracted. Secondarily, study findings (themes and/or categories) with illustrations (participants' voices) were extracted [42].

Included findings were synthesized using meta-aggregation [[40], [41], [42]]. Following extraction, study results were categorized based on conceptual similarities. Categorization was performed following a thorough and repeated examination of the original results. Each category described a key concept arisen form the aggregation of at least two original results and was accompanied by the most relevant and explanatory illustrations [42]. Similar categories were then aggregated into overarching synthesized findings, expressed as explanatory statements, based on which, recommendations for practice and research were developed [40].

The first author performed data extraction. Results categorization and synthesis were, on the contrary, discussed and their appropriateness confirmed by the whole research team, which included a peer-researcher. This procedure allowed multiple perspectives to be taken into account in the aggregation process, deepened the understanding of the phenomenon and enhanced the validity of the synthesis process.

### 2.7 Confidence in the findings

Confidence in the review findings was assessed following the Confidence in the Output of Qualitative research synthesis (ConQual) approach [46]. For each final synthesized finding, a confidence level was established based on the dependability and credibility scores of the included studies.

Dependability levels were estimated using the question #2, question #3, question #4, question #6 and question #7 of the Critical Appraisal Checklist [43]. All qualitative studies were first rated as “high” and were downgraded based on the answers to the five questions. If they obtained four to five “yes”, the rank did not change (high). With two to three “yes”, their rank was decreased by one level (from high to moderate). With zero to one “yes”, the rank was decreased by two levels (from high to low) [43,46]. Final synthesized findings were then downgraded based on the aggregate level of dependability of the included studies. If most of them scored “low”, the dependability of the findings was lowered by two levels and designated as “low”. If the majority scored “moderate” or a mix of “moderate” and “high”, dependability was downgraded one level and designated as “moderate”. If the majority scored as “high”, no downgrade was performed and dependability was designated as “high”.

Credibility evaluates the degree of “fit” between the authors’ interpretations and the supporting data [42,46]. Each result drawn from the included studies was evaluated and a level of credibility assigned. A finding was ranked as unequivocal (U) when the accompanying illustration was beyond reasonable doubt, equivocal (E) when the supporting illustration lacked a clear association with it and could be challenged, or unsupported (UN) when it was not supported by data. Final synthesized findings were further downgraded for credibility if not all the included results were unequivocal (one level down for a mix of unequivocal and equivocal findings; two levels down if all equivocal findings; three levels down for a mix of equivocal and unsupported findings; four levels down if all unsupported findings).

A confidence final score of high, moderate, low or very low was established for each synthesized finding.

## 3 Results

### 3.1 Literature search

**Fig. 1:**
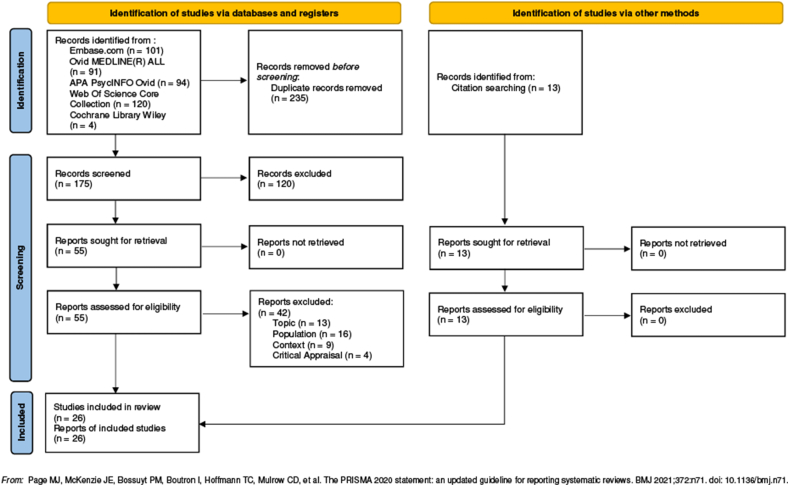
PRISMA flow diagram.

A total of 423 studies were identified through database search (n = 410) and other sources (n = 13). After duplicates removed, 188 papers were screened and 120 excluded based on title and abstract. The remaining 68 full-texts were assessed and 42 removed based on inclusion criteria. Four more studies were removed because participants’ voices were not adequately represented (question #8 of the Critical Appraisal Checklist). Finally, 26 studies were included in the synthesis (Fig. 1).

### 3.2 Critical appraisal of the included studies

Critical appraisal results are detailed in Table 1. Globally, 3 studies scored positively on all the items [[47], [48], [49]], 11 scored positively on seven to nine items [[50], [51], [52], [53], [54], [55], [56], [57], [58], [59], [60]] and 12 scored positively on six items or less [[61], [62], [63], [64], [65], [66], [67], [68], [69], [70], [71], [72]]. Researchers' philosophical perspective and its congruity with the research methodology was unclear in 16 out of 26 studies [52,55,57,58,[60], [61], [62], [63], [64], [65], [66],[68], [69], [70], [71], [72]]. In more than half of the studies the research methodology showed congruity with the research objectives [[47], [48], [49], [50], [51], [52], [53], [54], [55], [56], [57], [58], [59], [60]], the methods to collect data [[47], [48], [49], [50], [51], [52], [53], [54], [55], [56], [57], [58], [59], [60],^67^], the analysis of the data [[47], [48], [49], [50], [51], [52], [53], [54], [55], [56], [57],^59^,^60^,^67^] and the interpretation of the results [[47], [48], [49], [50], [51], [52], [53], [54], [55], [56], [57], [58], [59], [60]]. Among all included studies, only 4 addressed the influence of the researcher on the research [[47], [48], [49],^58^] and only 9 located the researcher culturally and theoretically [[47], [48], [49],^51^,^52^,^59^,^60^,^64^,^68^]. All the selected studies adequately represented participants’ voices and drew their conclusions from the analyses and interpretations of the data. Three studies did not report sufficient information on ethical approval [[61], [62], [63]].

**Table 1:** Critical appraisal of included studies.

Author(s)	Year of publication	Critical Appraisal Checklist									
		Q1	Q2	Q3	Q4	Q5	Q6	Q7	Q8	Q9	Q10
Andreasson and Skärsäter	2012	Y	Y	Y	Y	Y	N	N	Y	Y	Y
Bennett et al.	1993	U	U	U	U	U	N	N	Y	U	Y
Campbell	2008	U	U	U	U	U	N	N	Y	U	Y
Hughes et al.	2009	U	U	U	U	U	N	N	Y	U	Y
Johansson and Lundman	2002	Y	Y	Y	Y	Y	Y	Y	Y	Y	Y
Katsakou et al.	2011	U	U	U	U	U	Y	N	Y	Y	Y
Katsakou et al.	2012	Y	Y	Y	Y	Y	Y	N	Y	Y	Y
Klingemann et al.	2021	U	Y	Y	Y	Y	Y	N	Y	Y	Y
Kuosmanen et al.	2007	U	U	U	U	U	U	N	Y	Y	Y
Loft and Lavender	2016	Y	Y	Y	Y	Y	N	U	Y	Y	Y
Lorem et al.	2015	U	U	U	U	U	U	N	Y	Y	Y
McGuinness et al.	2013	Y	Y	Y	Y	Y	Y	Y	Y	Y	Y
McGuinness et al.	2018	Y	Y	Y	Y	Y	N	N	Y	Y	Y
Murphy et al.	2017	U	Y	Y	Y	Y	N	N	Y	Y	Y
Olofsson and Jacobsson	2001	Y	Y	Y	Y	Y	N	N	Y	Y	Y
Potthoff et al.	2022	U	Y	Y	Y	Y	Y	N	Y	Y	Y
Sibitz et al.	2011	Y	U	Y	Y	U	N	N	Y	Y	Y
Smyth et al.	2017	U	Y	Y	Y	Y	N	N	Y	Y	Y
Smyth et al.	2021	U	U	U	U	U	N	N	Y	Y	Y
Stylianidis et al.	2017	U	U	U	U	U	Y	U	Y	Y	Y
Terkelsen and Larsen	2013	U	Y	Y	U	Y	N	Y	Y	Y	Y
Valenti et al.	2013	Y	Y	Y	Y	Y	Y	N	Y	Y	Y
Verbeke et al.	2019	Y	Y	Y	Y	Y	Y	Y	Y	Y	Y
Wyder et al.	2015	U	U	U	U	U	N	N	Y	Y	Y
Wyder et al.	2015b	U	U	U	U	U	N	N	Y	Y	Y
Wyder et al.	2016	U	U	U	U	U	N	N	Y	Y	Y

Note: Q1 = Is there congruity between the stated philosophical perspective and the research methodology?; Q2 = Is there congruity between the research methodology and the research question or objectives?; Q3 = Is there congruity between the research methodology and the methods used to collect data?; Q4 = Is there congruity between the research methodology and the representation and analysis of data?; Q5 = Is there congruity between the research methodology and the interpretation of results?; Q6 = Is there a statement locating the researcher culturally or theoretically?; Q7 = Is the influence of the researcher on the research, and vice-versa, addressed?; Q8 = Are participants, and their voices, adequately represented?; Q9 = Is the research ethical according to current criteria or, for recent studies, and is there evidence of ethical approval by an appropriate body?; Q10 = Do the conclusions drawn in the research report flow from the analysis, or interpretation, of the data?; Y = yes; N = no; U = unclear.

### 3.3 Included studies

Study characteristics are presented in Table 2. Most of the papers reported on studies conducted in Europe (21 studies), especially in UK [51,53,59,[62], [63], [64]], Ireland [48,54,55,57,72] and Scandinavia [47,50,56,58,66]. One paper reported on a study conducted in USA [61] and three on a study in Australia [[69], [70], [71]]. One study was multicentred, including participants from Italy, Poland and UK [52]. All studies were published between 1993 and 2022, with 20 papers out of 26 being published after 2010 and 13 after 2014. The studies were all qualitative except for four, which used mixed methods [61,62,64,72]. The number of study participants ranged from 4 to 108. Study participants were mainly compulsorily admitted to hospital (21 studies). Two papers reported on studies including only voluntary patients [52,64] and one used a mixed sample of voluntary and involuntary patients [61]. In two papers, the admission status of the participants was not defined [49,65]. Data were mainly collected through interviews: semi-structured (19 studies), narrative (2 studies) and individual (1 study). Focus groups were used in three studies [57,62,68]. One study combined interviews and participant observation [58]. A great variety of methods was performed for data analysis, such as thematic analysis, content analysis, grounded theory and interpretative phenomenological analysis (IPA). Three papers did not specify the method of analysis applied [58,61,62].

**Table 2:** Characteristics of the included studies.

Author(s)	Year of publication	Country	Aim	Study methodology	Participants	Data collection method	Data analysis method
Andreasson and Skärsäter	2012	Sweden	To describe the experiences of care and treatment of patients involuntarily admitted to hospital	Phenomenography	12 psychotic patients with experience of involuntary treatment	Semi-structured interviews	Phenomenographic approach
Bennett et al.	1993	USA	To describe patients' perceptions of the morality of attempts made by others to influence them to be admitted to the hospital	Qualitative arm of a mixed-methods study	70 voluntary and involuntary psychiatric patients	Semi-structured interviews	Qualitative analysis
Campbell	2008	Northern Ireland	To explore the nature and quality of information, and legal advocacy services provided to patients and their carers during and after involuntary psychiatric hospitalisation	Qualitative arm of a mixed-methods study	4 groups of involuntarily admitted patients and 1 carer group* (44 participants in total)	Focus groups	Qualitative analysis
Hughes et al.	2009	UK	To provide a thorough patient perspective on involuntary hospitalisation, and on how it is perceived to have affected the self, relationships and recovery	Qualitative	12 participants with experience of involuntary admission	Semi-structured interviews	Thematic analysis
Johansson & Lundman	2002	Sweden	To describe the experience of being submitted to involuntary psychiatric care	Hermeneutic phenomenology	5 patients involuntarily admitted to a psychiatric hospital during the latest 2 years	Narrative interviews	Phenomenological hermeneutic analysis
Katsakou et al.	2011	England	To explore which experiences, according to patients, lead to feel coerced both at admission and during treatment	Qualitative arm of an exploratory mixed-methods study	36 voluntarily admitted patients	Semi-structured interviews	Thematic analysis
Katsakou et al.	2012	England	To explore the retrospective views of involuntarily admitted patients' on why their hospitalisation was right or wrong	Grounded theory	59 involuntarily admitted patients	Semi-structured interviews	Grounded theory
Klingemann et al.	2021	United Kingdom, Italy and Poland	To explore treatment pressures put on patients by clinicians and by patients' relatives, during formally voluntary admission to psychiatric hospitals	Theory-driven qualitative	108 voluntarily admitted patients	Semi-structured interviews	Theoretical thematic analysis
Kuosmanen et al.	2007	Finland	To explore patients' views about whether they were deprived of their liberty during psychiatric hospitalisation	Qualitative explorative	51 inpatients of two acute psychiatric wards	Semi-structured interviews	Inductive content analysis
Loft and Lavender	2016	UK	To explore compulsory admission experiences of patients with psychosis, and identify their key characteristics	Grounded theory	8 service-users with experience of two or more involuntary admission due to psychotic symptoms and 9 psychiatrists*	Semi-structured interviews	Grounded theory
Lorem et al.	2015	Norway	To explore the patients experiences of coercive measures and their description of the elements that determine how they were ‘morally’ evaluated	Qualitative inductive	5 patients with various experiences of coercion	Individual interviews	“Intuitive” approach and thematic analysis
McGuinness et al.	2013	Ireland	To explore the impact and the lived experience of involuntary hospital admission	Phenomenology	6 patients involuntarily admitted to a psychiatric hospital	Semi-structured interviews	Interpretative Phenomenological Analysis (IPA)
McGuinness et al.	2018	Ireland	To develop a theoretical framework for understanding patients' experiences of involuntary hospitalisation	Grounded theory	50 patients involuntarily admitted to hospital	Semi-structured interviews	Grounded theory and Constant comparative method
Murphy et al.	2017	Ireland	To explore patients' experiences throughout their involuntary admission	Qualitative descriptive	50 patients involuntarily admitted to hospital	Semi-structured interviews	Thematic analysis
Olofsson and Jacobsson	2001	Sweden	To describe involuntarily admitted patients' experience of coercion, and their views on how to prevent coercion	Narrative	18 involuntarily admitted psychiatric patients	Narrative interviews	Qualitative interpretative content analysis
Potthoff et al.	2022	Germany	To develop a conceptual framework of psychological pressure based on patients experience during inpatient stays	Grounded theory	14 mental healthcare service users with previous experience of involuntary hospital admission	Semi-structured interviews	Grounded theory
Sibitz et al.	2011	Austria	To establish a typology of coercion perspectives and styles of integration into life stories	Modified grounded theory	15 participants with a history of involuntary hospitalisation	Semi-structured interviews	Thematic content analysis
Smyth et al.	2017	Ireland	To explore the perspectives of key stakeholders involved in the involuntary admission and detention of people under the MHA 2001	Qualitative descriptive	5 patients with experience of involuntary hospitalisation, 8 relatives* and 49 members of other stakeholder groups*	Focus groups	General inductive approach
Smyth et al.	2021	Ireland	To examine and compare perceptions of patients about their involuntary hospitalisation with levels of insight	Mixed method design	42 participants, three months after being discharged from involuntary admission	Semi-structured interviews	Content analysis
Stylianidis et al.	2017	Greece	To investigate patients' perspectives on involuntary hospitalisation	Qualitative interpretative	14 involuntarily hospitalized patients one month after discharge	Focus groups	Interpretative thematic analysis
Terkelsen and Larsen	2013	Norway	To explore how health professionals and patients act and describe their experiences of involuntary admission into a locked ward	Ethnography	16 involuntary psychiatric inpatients (4 interviewed), 22 health professionals (18 interviewed)*	Participant observation and interviews	Qualitative analysis
Valenti et al.	2013	England	To explore the perceptions of involuntarily admitted patients about situations occurring in the hospital.	Moral deliberative	59 patients involuntarily admitted to acute wards	Semi-structured interviews	Thematic content analysis
Verbeke et al.	2019	Belgium	To propose a model of the relational elements of coercion based on patients' assumptions	Interpretative phenomenological analysis (IPA)	12 hospitalized psychiatric patients	Semi-structured interviews	Interpretative phenomenological analysis (IPA)
Wyder et al.	2015	Australia	To explore the interactions between involuntarily admitted patients' and health care professionals within an acute mental health ward	Qualitative interpretative	25 patients hospitalized under Involuntary Treatment Order (ITO)	Semi-structured interviews	General inductive approach
Wyder et al.	2015b	Australia	To analyse the patients' experiences and understandings of the legal process of an involuntary treatment order	Qualitative interpretative	25 patients hospitalized under Involuntary Treatment Order (ITO)	Semi-structured interviews	General inductive approach
Wyder et al.	2016	Australia	To explore the tensions between the principles of empowerment and control, and involuntary treatment	Qualitative interpretative	25 patients hospitalized under Involuntary Treatment Order (ITO)	Semi-structured interviews	General inductive approach

Note: *Excluded from the synthesis.

### 3.4 Meta-aggregation findings

Totally, 151 findings were extracted from the 26 studies and included in the synthesis (Table 3). All findings were rated as unequivocal. These findings were aggregated into 27 categories and 7 synthesized findings (Supplementary file 2). Synthesized finding 1 focus on the patients’ experience of hospitalisation and the associated feeling of coercion. Synthesized findings 2, 3 and 4 describe the factors affecting their perception of coercion. Synthesized findings 5, 6 and 7 explore respectively the coping strategies adopted by patients to deal with the feeling of being coerced, the impact of the experience on their lives and their suggestions for alternatives.

**Table 3:** Study findings: original themes and subthemes.

Author(s)	Year of publication	Original themes and subthemes
Andreasson and Skärsäter	2012	Receiving needed support Receiving good care (U) Receiving needed shelter (U) Receiving help with understanding (U) Receiving care in a healing setting(U) Perceiving respectful care Acknowledgment as a human being (U) To be independent (U) To participate (U)
Bennett et al.	1993	Inclusion (U) Beneficent motivation Evaluation of others' motives (U) Effects of evaluation of others' motives (U) Good faith Qualifications: formal and informal (U) Deceit (U) Respect (U)
Campbell	2008	Experiences of compulsory admission (U) Information and advice following compulsory admission (U)
Hughes et al.	2009	During involuntary hospitalisation Views of self (U) Experience of relationships and interactions (U) Medication (U)
Johansson and Lundman	2002	Being restricted in autonomy (U) Being violated by intrusion on physical integrity and human value (U) Being outside and not seen or heard (U) Being respected as an individual (U) Being protected and cared for (U)
Katsakou et al.	2011	Experiences leading to perceived coercion Hospital treatment not effective/need for alternative treatment (U) Not participating sufficiently in the admission and treatment process (U) Not feeling respected/cared for (U) Experiences not leading to perceived coercion Need for hospital treatment and safety (U) Participating in the admission and treatment process (U) Feeling respected/cared for (U)
Katsakou et al.	2012	Common experiences between groups: Mentally unwell/at risk before admission (U) Feeling out of control during hospitalisation (U) Positive group: Need for coercive intervention: not recognising problems when unwell (U) Averting risk and feeling safe in hospital (U) “Negative” group: Need for non-coercive treatment (U) Unjust infringement of autonomy (U) Ambivalent group (U)
Klingemann et al.	2021	Treatment pressures Persuasion (U) Interpersonal leverage (U) Informal coercion Threat (U) Someone else's decision (U) Violence (U)
Kuosmanen et al.	2007	Type of deprivation of liberty used Restrictions on leaving the ward (U) Restrictions on communication (U) Coercive measures (U) Confiscation of property (U) Patients' feelings about deprivation of their liberty (U) Reasons for deprivation of liberty as perceived by patients (U)
Loft and Lavender	2016	Deteriorating mental health of service user (U) Professionals remove service user's liberty (U) Managing mental health on the psychiatric ward (U) Regaining liberty (U) Recovery in the community (U)
Lorem et al.	2015	Agreeing and accepting Coercion seen as help and care (U) Trust in health personnel (U) Fighting or resisting Physical resistance (U) Appeal to patient rights (U) Resignation Lack of information or good reason (U) Not being heard or no opinions (U) Regulation and informal coercion (U) Excessive or unnecessary use of force (U)
McGuinness et al.	2013	The early days (U) Experiences of treatment (U) Moving on? (U)
McGuinness et al.	2018	Theory of preserving control (ToPC): Losing control Diminishing self-mastery (U) Feeling violates (U) Being confined (U) Regaining control Resisting system (U) Encountering humanising care (U) Gaining perspective (U) Playing ball (U) Maintaining control Living with the consequences of involuntary hospitalisation (U) Managing mental health (U) Preserving sense of self (U)
Murphy et al.	2017	Feeling trapped and coerced (U) Lack of informational and emotional support (U) Admission-induced trauma (U) Person-centered encounters (U)
Olofsson and Jacobsson	2001	Not being respected as a human being Not being involved in one's own care (U) Receiving care perceived as meaningless and not good (U) Being an inferior kind of human being (U) Being respected as a human being Being involved in one's own care (U) Receiving good care (U) Being a human being like other people (U) Respecting the staff (U) Alternatives to coercion Coercion was necessary as protection (U) Preventing coercion lasting too long (U) Voluntary admittance (U) Outpatient care (U) Human contact and handling problems (U)
Potthoff et al.	2022	Aims of communication Pressure to improve adherence to recommended treatment (U) Pressure to improve adherence to social norms (U) Ways of communicating Explicit statements (U) Nonverbal communication (U) Things that go unsaid (U) Contexts of communication The quality of the personal relationship (U) The institutional setting (U) The material surroundings (U) Convergence between the parties' understanding of mental disorder (U)
Sibitz et al.	2011	Perspectives on involuntary admission and coercion A necessary emergency brake (U) An unnecessary overreaction (U) A practice in need of improvement (U) Integration of experiences into life stories Over, not to be recalled (U) A life-changing experience: 1. Impact on self-esteem and sense of self (U) 2. Impact on relationships and community life (U) 3. Impact on health (U) 4. Positive changes (U) Motivation for political engagement (U)
Smyth et al.	2017	Getting help (U) Detention under the Act Signing the application and the perceived impact on relationships (U) Information about the detention process (U) The need for therapeutic care (U) Experiences of the tribunal process *
Smyth et al.	2021	Understanding The need for involuntary admission (U) Perception of the impact of admission and diagnosis (U) The necessity of treatment (U) Emotional state (U) Humanising care (U)
Stylianidis et al.	2017	Views about involuntary hospitalisation Benefits (U) Ambivalence on justification (U) Experience of involuntary hospitalisation Negative emotions (U) Relationship with the staff (U) Relationship with families (U) Therapy versus oppression (U) Human devaluation (U) Dearth of knowledge and advocacy (U) Interventions and alternative suggestions (U)
Terkelsen and Larsen	2013	The ward as a hotel (U) The ward as a detention camp (U)
Valenti et al.	2013	Lack of control about decision making in the hospital: freedom (U) Benefits of involuntary treatment in terms of risk reduction: safety (U) Considering, listening and care in personal regard: respect (U)
Verbeke et al.	2019	Segregation Exclusively seen as a patient (U) Us and them (U) De-subjectivation Patients (U) Staff (U) Power resides in interactions Broken contact (U) Captured in silence (U) Conforming (U) Positive encounters (U)
Wyder et al.	2015	Staff potential to impact on ITO and hospital experiences (U) What are good relationships? Feeling connected (U) Finding time despite the busyness of the ward (U) Provision of information about the ward rules (U) Provision of information about ITO conditions (U) Being able to look beyond the illness (U) Relationships based on partnerships (U)
Wyder et al.	2015b	Experiences of the ITO The ITO protects from harm (U) ITO was experienced as an intrusion into their liberty and physical integrity (U) The mixed group (U)
Wyder et al.	2016	Overall experience of the ITO* ITO protected them from harm ITO is an intrusion on my liberties Factors affecting sense of agency Having a safe space to reflect on their experience (U) Understanding their ITO conditions and the ward expectations (U) Having input into their treatment (U)

Note: U = unequivocal; E = equivocal; UN = unsupported. *Excluded from the synthesis.

#### 3.4.1 Synthesized finding 1: patients perceived the hospitalisation and its restrictions either as a necessary form of protection or as a violation of their autonomy

This finding synthesized 52 different results extracted from 20 papers. Five categories were included in this synthesized finding: “being violated and losing control”, “being protected and cared for”, “feeling ambivalent”, “feeling unwell” and “type of coercion”.

Acknowledging that their situation before hospital admission was critical and that they were feeling unwell and losing control [51,53,54,60], some patients perceived the hospitalisation and its restrictions as a necessary form of protection and care [47,51,54,[56], [57], [58], [59],^63^,[66], [67], [68],^71^,^72^]:

*“Losing control is a double-edged sword. It’s losing functions which are necessary for healing. I was relieved of the responsibility. They saw that I could take no more. It’s like a mother who takes over when you don’t have any more in you. You become a child again; you have a similar emotional register, the feeling that there’s nobody out there. It’s negligence to do nothing; it would have been a new betrayal for me who had no parents to take care of me. It was, in fact, my first encounter with care. I really felt cared for.”* [66]

These patients described the hospitalisation and the treatment received, even though under coercion, as the only possible solution because of their inability to recognise their need for help during crisis [51,54,57,67,72]:

*“I was becoming extremely anxious and psychotic, so I did need to be taken into hospital…Once it goes past a certain point, I don’t understand the processing, I don’t understand why I need to go into hospital…so I think I did need to be taken under section because I don’t think I’d have agreed voluntarily …”* [51]

Thus, patients experienced admission to hospital as essential to guarantee their own safety and the safety of others [47,51,54,56,59,[66], [67], [68],^71^,^72^]:

*“Yeah…because of my safety, my safety and other people’s safety,* [was more important] *to keep me safe than let me have my freedom”* [59]

Beside protection, these patients also recognised that the hospitalisation and treatment had a positive impact on their mental health and social situation, giving them the opportunity to take a break, rest, recover and gain perspective on their situation [47,51,54,63,71,72]:

*“I felt relieved because I was exhausted…I was delighted I could get a bit of rest…I was delighted when I arrived there …”* [72]

These feelings sometimes even led patients to perceive involuntary care as voluntary [47,58]:

*“… afterwards I don't experience it as directly coercive…I just think that if you have no insight into your illness then…you get it sooner or later and then you don't experience it as coercion* [the involuntary care].” [47]

On the other hand, patients recognised that various forms of coercion were enacted on them during hospitalisation, through both formal measures and informal pressures [52,60,65,66]. As a result, most of them described the hospital experience as a violation of their liberty leading to the subsequent feeling of loss of control [47,51,[53], [54], [55],[57], [58], [59],^62^,^63^,[65], [66], [67], [68],^71^,^72^]:

*“All your rights are taken away, it’s horrible, you are not in control anymore.”* [59]

These patients often experienced admission to hospital as a brutal disruption of their lives and an infringement of their rights and physical integrity. Indeed, an unnecessary use of force and violence, a recurrent involvement of the police and use of handcuffs during the admission process *“Like we are some kind of criminals”* [68] as well as the excessive use of medication once in hospital were frequently reported [51,54,55,[57], [58], [59],^62^,^63^,^68^,^71^,^72^]:

*“As soon as you get in there they give you medication … and basically if you refuse too many times they put you in what they call the lock, the proper lock-up.”* [59]

Patients felt overpowered by the staff, patronized by their attitude and by the inflexibility of the ward rules, which caused them to perceive a total loss of power [47,51,54,55,58,59,65,66,71]:

*“You got no rights. You go to bed when they say. If you don't go to bed, they'll give you drugs. You wake up when they say. You see a doctor when they say. And you don't have any rights.”* [71]

The perception of violation and loss of control was often accompanied by anger, sadness, anxiety, distress, fear and terror to the extent that some patients described the experience of hospitalisation as traumatic [54,55,57,62,63,65,67,68,71,72]:

*“They took me back to the room, they put me face down on the bed, actually holding my face into the cushions, so that I couldn’t breathe. I was fighting and fighting. And they were saying, um, go on, pull her trousers down and stick it in her arse. I thought they were raping me.”* [63]

The only way to regain liberty and control was to be discharged [53] and never come back [68]. However, for some patients, the feeling of not being in control anymore continue after discharge [51]:

*“Once you’ve been in hospital if they say you’ve got to go into hospital, you have got to go; like being under the surgeon’s knife: once under the surgeon’s knife, always under the surgeon’s knife.”* [51]

Finally, for a small group of patients, this dilemma between protection and violation remained unresolved leading them to express ambivalent feelings towards the experience of hospitalisation and its restrictions [48,51,71,72]:

*“You do feel like you have your rights taken away from you…like a second class citizen…but ….maybe deep down knowing…it can be good for you is the way I look back on it.”* [72]

#### 3.4.2 Synthesized finding 2: patients perceived coercion when they lacked involvement in the decision-making process

This finding synthesized 35 different results extracted from 20 papers. Three categories were included in this synthesized finding: “being informed”, “participating in care” and “preserving autonomy”.

Patients’ perception of coercion was strongly affected by the feeling of being heard and involved in the decision-making process throughout the admission. In order to feel involved in decision-making, patients needed first to be informed [[47], [48], [49], [50], [51],[55], [56], [57],^60^,^62^,[66], [67], [68], [69], [70],^72^]. Patients frequently raised need for information as an important issue. Indeed, many claimed that poor information was provided about the treatment they were asked to follow as well as the hospital functioning and rules, leading them to feel objectified and disregarded [[47], [48], [49], [50],^56^,^60^,^67^,^70^]:

*“When I asked for information at about the drug itself they’d only give me the printed leaflet from the pharmaceutical company…I wanted more and I wanted access to some other form of info but I was never allowed it.”* [48]

The dearth of knowledge about the reasons, duration and course of the admission or the coercive measures they were submitted to, as well as about their rights while under coercion, increased the patients’ feeling of loss of control and helplessness [48,51,[55], [56], [57],^62^,[66], [67], [68], [69],^72^]:

*“They never told me why I was sectioned, it’s like taking you and locking you up, never telling you why you are being locked up! I felt like a prisoner!”* [51]

Beside information, most patients also wanted to have a say in their own treatment [47,48,50,54,56,59,61,64,66,69,70]:

*“I want a say in my health, I want a say in what medication I’m on. I want to know the side effects before they put me on drugs.”* [70]

However, they often reported that no choices were offered to them, hindering their active participation in care [48,50,64]:

*“I didn't really decide, they decided for me…I thought that if I didn't say yes then I would be sectioned, so really I did feel coerced…it certainly didn't feel like I had a choice, so I got angry.”* [64]

Although not all patients required the same degree of participation, they all wanted to be heard and taken into account by the staff [54,56,61,66,69]. Patients would like professionals to recognise their expertise and knowledge, gained from direct experience of the disease, as a valuable source of information:

*“They* [the doctors] *should ask you what medication is working for you. Who else would know! I told them that I felt I was on too many medications, but they would not listen. It is a mental drag. It could be the way I was expressing myself, as I find it difficult to find my words. Medication is one of the things the patient knows, because they are the ones going through it. I just wanted them to listen to my concerns about the medication, but they wouldn’t.”* [69]

Co-operation, partnership and shared decision-making were identified as useful tools to meet this need and improve patients’ feeling of being actively involved in their treatment [47,54,59,69]:

*“When they sat down whit me and…we done the Care Plan together. That is the only time out of all my admissions…It does make a difference, because then I have some say in it, in the Care Plan.”* [59]

Maintaining a certain degree of responsibility and autonomy, even though under coercion, also helped patients to feel less constrained and regain some sense of agency and control [47,49,50,54,59,66]:

*“Even in situations where coercion is used against me, I demand some degree of influence.”* [66]

When no room was granted to self-determination, patients felt disempowered, with a resulting increased feeling of coercion [49]:

*“I felt as if I were a child in a boarding school, you can't decide anything for yourself. Not even if you want chocolate paste or cheese on your sandwich, that's really absurd.”* [49]

#### *3.4.3* *Synthesized finding 3:* patients perceived coercion when they experienced unsupportive, disrespectful and unreliable relationships

This finding synthesized 42 results extracted from 18 papers. Four categories were included in this synthesized finding: “feeling respected and treated fairly”, “being in contact with staff and getting support”, “trusting the other” and “interacting with family, friends and other patients”.

Relationships play a key role in the patients’ experience of coercion during hospitalisation, especially those with the healthcare professionals. First, in order to perceive less coercion patients needed to feel that the staff treated them respectfully and fairly [[47], [48], [49], [50],[54], [55], [56],[59], [60], [61],^64^,[67], [68], [69]]:

*“To be honest, it was just like me walking in myself. That’s how it felt. It didn’t feel bad. It really didn’t, in fairness. I was treated just like any other person that would walk in off the street, I’d say. They weren’t bad-minded to me or talk down to me or, they just treated me like a normal person, which was good, you know.”* [55]

Indeed, patients often expressed a desire to be taken seriously, to be valued as human beings and treated as any other person, in spite of their condition [[47], [48], [49], [50],[54], [55], [56],^60^,^61^,^64^,[67], [68], [69]]:

*“… everybody ought to be valued equally even if you are ill. But sometimes you are made to feel that you are not worth anything, have no human value. But most of them* [the staff] *are nowadays I suppose, they are fellow human beings as they should be.”* [47]

When this did not happen, patients felt deprived of their dignity and objectified by the healthcare professionals [[47], [48], [49],^56^,^61^,^64^,^67^,^69^]:

*“You become a nobody, they can do whatever they want with you, although maybe you are a very valuable person being in a crisis.”* [56]

Disrespect was also perceived when the limits of physical integrity were breached or treatment imposed with force [47,56,59,68]:

*“Disrespectful … They take you with push or whatever.”* [59]

Furthermore, patients perceived less coercion if they encountered professionals who were empathetic, committed and supportive towards them [[47], [48], [49], [50],[54], [55], [56], [57],^60^,^63^,[67], [68], [69],^72^]:

*“I suppose it started off with the Gardaí* [Irish police]*, and they took a very caring attitude. They seemed concerned…When I got here* [hospital] *then, it was the nurse that actually brought me in, that kind of ran me through everything that kind of signed me in to the ward. It was caring as well. She was actually talking to me…I just think that engagement in itself helped relax me.”* [55]

Patients reported that what they especially needed from staff was their closeness. They wanted someone to talk to, someone who cared about them and who took time to listen to them. However, often professionals were perceived as unavailable or lacking real interest in them [47,48,50,[54], [55], [56],[67], [68], [69],^72^]:

*“The worst thing you can hear from the person who decides over your life and treatment is that he/she does not have time for you.”* [56]

*“What do mental people who are going through a very hard time need? Kindness…A touch of reassurance…I would have preferred a cancer diagnosis than that because my fear of all of that…There was no hint of ‘look, we really want to help you …”* [72]

Closeness with professionals was also hindered by the inflexible and authoritarian application of hospital procedures and rules, which in extreme cases could appear as real punishments in the eyes of patients [47,49,55,63]:

*“They used to take away my furniture, so I was left with a mattress on the floor. And, no sheets, no bedding [ …] So those were the ways that they used to punish me.”* [63]

Feeling respected and supported improved patients’ trust towards the healthcare staff, another key element in reducing the perception of coercion during hospitalisation [49,60,61,66,69]. In order to trust professionals and the other persons taking part to the admission process, patients must feel that their choices were driven by a sincere concern for them and taken in their best interest:

*“How did you feel about that?* [a friend becoming involved in the hospitalization decision when the patient threatened to hurt herself] *P: I was happy. Because nobody ever cared enough about me to do that…Because he heard what I had to say. He wasn’t all right with me attempting to do what I had to do. He told me my life was worth something.”* [61]

Finally, even though on a smaller scale, interactions with family members, friends and other patients were also mentioned as having an impact on patients’ experience of coercion during hospital admission [47,60,63,68]. While in some cases, they represented a positive resource, for other patients they were an additional barrier to their autonomy and a source of coercion:

*“But I made very good contact with the other patients, so that was very nice. We supported one another …”* [47]

*“They lie…they* [the doctors] *believe the family members* (…)*. And when the family members speak to the doctors, the doctors never talk to the patient, they don’t believe the patient.”* [68]

#### 3.4.4 Synthesized finding 4: patients perceived coercion when they experienced hospital treatment as ineffective and unsafe

This finding synthesized 12 results extracted from 10 papers. Two categories were included in this synthesized finding: “hospital perceived as effective” and “hospital perceived as a safe place”.

The perception of the hospital treatment as effective or useless, even in retrospect, may influence the overall experience of hospitalisation and the associated feeling of coercion [50,51,53,54,56,60,64,68,70,72]:

*“The main helpful part of the sectioning was to get me back onto treatment. Take me out of the community for a while and get me back on my medication, back on my treatment.”* [51]

While some people recognised that hospital helped them to get better, for others it was the wrong answer to their problems, which even worsened after admission [51,53,54,60,64,68,72]:

*“I had gone into the ward feeling bad and it just made me feel worse I guess. I mean perhaps it was the right place for me but it didn't seem like anything was being done at the time. It seemed odd being in hospital and not seeing any sort of treatment at all.”* [64]

Activities and psychotherapy were valued as useful care tools, but unfortunately often lacking, while medication was the main and, in many cases, the only treatment available [50,53,54,56,64,70,72]:

*“This is a place where you’re supposed to go to become better ... in reality, it’s a place where you’re forced to take medication and you can go …”* [53]

*“I just felt that I was having medication thrown at me…there was a lot of psychological aspects to it that aren’t really addressed. Everyone seemed to be focusing on drugs as a solution.”* [72]

In order to perceive less coercion, patients also needed to feel safe while in hospital. As already mentioned above, some patients were aware of their critical situation before admission and thus perceived the hospitalisation and its restrictions as a necessity. In these cases, the hospital was experienced as a safe place that provided them with the needed protection from the outside world and the consequences of their illness [51,54,60,64,70]:

*“I was feeling that people were following me, watching my every move. To me, the only safe place I felt was the psychiatric hospital.”* [64]

In contrast, others reported being concerned about their safety during their stay, mainly due to the condition of other patients and to the unsettling environment [53,70]:

*“Some people continue to take* [illicit] *drugs on the ward...it’s quite easy to get drugs in there …”* [53]

#### 3.4.5 Synthesized finding 5: when they felt coerced, patients resorted to various coping strategies to deal with the situation

This finding synthesized 12 results extracted from 8 papers. Five categories were included in this synthesized finding: “acknowledging and agreeing”, “conforming”, “resisting”, “resigning” and “moving on”.

When facing hospitalisation and its restrictions, patients resorted to different coping strategies. Those who experienced the hospitalisation as the only possible way to guarantee their safety and ensure their well-being were more inclined to interpret the restrictions they were subjected to as justified on medical and safety grounds and therefore to acknowledge them [65,66]:

*“… I understand that a hospital has to have rules... … restrictions are based on medical reasons... … rules are OK …”* [65]

When restrictions were implemented by professionals with whom patients had a positive relationship of cooperation and trust, the existence of conflicting positions was more acceptable and agreement was fostered [66]:

*“Olav* [the medical doctor] *and I have always agreed. Although he insisted on his view and I insisted on mine, we could find solutions. It’s one of the reasons why I trust him blindly as a professional, and he trusts me, and it’s a very good thing, because I feel I have a friend for life there. So it’s about human relations.”* [66]

Other patients did not accept the situation, but conformed to the system in order to avoid or at least minimise coercion and be discharged more quickly [48,49,54,72]. Conforming is often the result of a learning process that patients carried out through their inpatient experiences. These patients have learnt to monitor constantly what they say and how they behave, and to never disagree with professionals. Playing the role of the “good patient” is thus a form of self-coercion enacted to cope with the situation:

*“I was kind of agreeing and nodding with everything just to get through … I’m thinking to myself…you…shut your mouth and go along with it … and hopefully get out fast…One of the patients said to me when I got in…you agree with everything. You say yes to everything, you toe the line or else.”* [54]

When hospitalisation and its restrictions were experienced as an unjust violation, patients’ main reaction was to fight back and resist [54,66,72]. Opposition could be expressed through violation of hospital rules, verbal confrontation or physical resistance:

*“Because of the aggression of the admission, the aggression in me wanted to fight … I was angry…I was just fighting back to prove to them that I’m all right. I didn’t need this sort of intervention.”* [54]

However, many patients soon realised that resisting was pointless and therefore moved on to resignation [56,66,72]. Resigning was seen as the only way to deal with a situation in which they were left with no real alternatives or choices:

*“They had somehow got legal authority to forcibly medicate me, and they would certainly do that, and then suddenly one of the doctors said: ‘We’ve entered into an agreement, either belts or medication’. So I said it was like choosing between plague and cholera, and that an agreement cannot be entered into by force…and then they were about to put me in belts, but I managed to avoid it by taking medicine.”* [66]

Finally, for some patients, the only way to cope with this experience was to distance themselves from it, remove it from their memory and never speak of it again once it was over [48,67]:

*“I don’t want to talk about or remember it, and when I realise in a dialogue that the other person is affected as well and might find it burdensome, then it is even worse ...usually I don’t think about it anymore because I don’t want to remember, same with regular psychiatric hospitalisations.”* [67]

#### 3.4.6 Synthesized finding 6: when perceived as coercive, the experience of hospitalisation negatively affected several areas of patients’ identity and life

This finding synthesized 14 results extracted from 8 papers. Four categories were included in this synthesized finding: “well-being and mental health”, “relationships and social life”, “view of self” and “activities and daily life”.

If perceived as coercive, the hospital admission negatively affected several areas of patients’ lives. Firstly, although some patients recognised that the hospitalisation had positive effects on them and helped them to gain insight on their condition [53,67], others reported that this experience did not make them feel better and on the contrary even worsened their health [54,55,67]. Some developed post-traumatic stress disorder symptoms and needed professional help to recover:

*“Leaving the hospital…that’s even worse, because that’s when the trauma comes in and the fear comes into your normal life. You have to go to work and keep living this like big trauma caused by these people* [involved in involuntary admission experience]*, and this trauma is the one that’s going to cause more severe and more problems.”* [55]

Due to the total loss of power and control experienced during hospitalisation, people subsequently felt vulnerable. Depression, anxiety and fear of reoccurrence of the experience were reported [55,67]:

*“I suppose it’s the fear of the reoccurrence of it* [involuntary admission]*. The fear of the fact that this abuse or whatever can happen again. That other people can decide how well I am without me expressing it. Other people can take charge, you know.”* [55]

The experience of coercion could be traumatising not only for patients but also for their relatives [51]:

*“The effect of me being sectioned was catastrophic to myself and my children. Social services had my daughter … they wouldn’t let me know where she was, because I’ve got a mental health problem. My son had absolutely gone ballistic. He was now in hospital. My other daughter was told I wasn’t coming back for six months after also being told that I was running up and down the motorway. She was put through an extreme amount of unnecessary stress. I wasn’t even running up the motorway for a start. That didn’t happen …”* [51]

Moreover, patients were often referred to hospital by a family member or a friend, who also signed the application form in case of coercion. This caused patients to lose confidence in their relatives and their relationships with them to deteriorate [54,57,67]:

*“I cannot forget that* [being signed in] *very easily. I felt very betrayed by my wife…I can’t trust her any more…Obviously it has affected my relationship…That made me a very disillusioned person.”* [54]

Patients’ social life was also strongly affected by the experience of coercion [51,53,54,57,63,67]. Social stigma and discrimination as well as the feeling of being treated differently after admission were reported [51,53,54,67]:

*“It* [point of removal] *was only 9 o’clock. There were people on the street…that seen all this happening which was … very embarrassing…people judge you as well on that actual admission or involuntary admission. There’s a stigma with it no matter what anybody says.”* [54]

In some cases, the negative experience offered the motivation to publicly engage with other service users and try to change the system [67]:

*“I said, ‘‘somewhere down the road, I will publicly take them to court’’ ...all of the hospitals, some of the doctors who treated me against my will and didn’t want to hear me…not to seek revenge but to draw attention to this issue and in light of the future for many other psychiatric users, who could experience the same thing, not being heard like I was.”* [67]

However, more frequently they reported that the hospital experience had severely affected their identity and psychological integrity, engendering a loss self-esteem and self-confidence, and making them feel worthless and insecure [54,55,63,67,72]:

*“I had no self-respect when I left there whatsoever*.” [63]

*“It leads to an absolute inferiority complex, I have the feeling that I am not worth talking to other people, already thinking that I am not worth it, well, we can say destroying my personality.”* [67]

Finally, hospitalisation was experienced as a disruption of patients’ life, affecting their daily habits and working life [51,67]. Long-term economic and legal consequences were therefore highlighted:

*“I don't find it good that it is on record…I can't get life insurance…and my driving licence is limited in time and to prolong it costs a lot of money.”* [67]

#### 3.4.7 Synthesized finding 7: patients called for less coercive and more effective alternative interventions when in a crisis

This finding synthesized 8 results extracted from 5 papers. Four categories were included in this synthesized finding: “outpatient services and mobile teams”, “human contact with professionals and other patients”, “voluntary admission and shorter coercive measures” and “personal strategies”.

Patients perceived a variety of alternatives as less coercive and thus more helpful when in crisis. Some stated that they would have preferred to be treated in their own environment instead of in hospital [51,68]:

*“There should be a way to be treated at home.”* [68]

Outpatient services and mobile teams, such as home treatment teams, crisis intervention teams and Assertive Community Treatment teams, were often mentioned as effective alternatives, as well as psychotherapy and counselling [51,54,56,67,68]:

*“I know this example about a team that intervenes in situations of crises, a good team, that convinces, that the police is not needed to convince people to go to the hospital and there are data saying that people recover faster after that, and it would pay to have such a team, with trust, and they explain why it is necessary to go to the hospital.”* [67]

For some patients, human contact and closeness with professionals, relatives and other patients sharing the same experiences were the only things they needed when in crisis [56,67,68]:

*“What I need is not only medication. I need my family’s care and support and to be close to friends and neighbours. I need other people to speak out and to share with me similar painful experiences.”* [68]

When hospitalisation was necessary, it had to be voluntary and based on non-coercive treatments and, if coercive measures could not be avoided, they had to last as short as possible [51,56,68]:

*“I said to the nurses they could call for the physician on duty to come and take away the extra supervision, as I felt fit then. If I am going to have extra supervision when I feel fit then I will soon be depressed again.”* [56]

Finally, some personal strategies, such as sleeping long hours, listening to music or writing, were also reported as effective non-coercive alternatives to deal with crisis [51,54]:

*“… a way I have dealt with a lot of things is to write things down…that for me has been very helpful, to write down my experiences…my emotions and my feelings and that’s something you know I can look back on and understand …”* [54]

### 3.5 Confidence of the synthesized findings

Confidence scores of the synthesized findings are presented in Table 4. All synthesized findings reached an overall confidence score of “moderate”. The seven findings were downgraded one level due to dependability limitations. No downgrade was needed on credibility since all extracted findings were rated as unequivocal.

**Table 4:** Confidence of the synthesized findings.

Synthesized findings	Dependability	Credibility	Overall score
1. Patients perceived the hospitalisation and its restrictions either as a necessary form of protection or as a violation of their autonomy	Downgrade 1 level	No downgrade	Moderate
2. Patients perceived coercion when they lacked involvement in the decision-making process	Downgrade 1 level	No downgrade	Moderate
3. Patients perceived coercion when they experienced unsupportive, disrespectful and unreliable relationships	Downgrade 1 level	No downgrade	Moderate
4. Patients perceived coercion when they experienced hospital treatment as ineffective and unsafe	Downgrade 1 level	No downgrade	Moderate
5. When they felt coerced, patients resorted to various coping strategies to deal with the situation	Downgrade 1 level	No downgrade	Moderate
6. When perceived as coercive, the experience of hospitalisation negatively affected several areas of patients' identity and life	Downgrade 1 level	No downgrade	Moderate
7. Patients called for less coercive and more effective alternative interventions when in a crisis situation	Downgrade 1 level	No downgrade	Moderate

## 4 Discussion

This review provided a thorough understanding of patients' experience of coercion during hospital admission. Seven synthesized findings were identified from 26 studies, depicting the patients' experience of coercion. Patients described the hospitalisation and its restrictions either as a form of care and protection against the negative effects of their illness, or as a violation of their rights and autonomy, leading to a feeling of loss of control and powerlessness (synthesized finding 1). Involvement in the decision-making process (synthesized finding 2); relationships with staff (synthesized finding 3) and satisfaction with hospital treatment (synthesized finding 4) showed to play a key role in patients’ experience of admission. Several coping strategies were deployed in order to deal with the feeling of being coerced (synthesized finding 5). Nonetheless, patients reported significant negative consequences in several areas of their lives (synthesized finding 6) and called for the implementation of less coercive and more effective alternatives (synthesized finding 7).

### 4.1 Synthesized finding 1: patients perceived the hospitalisation and its restrictions either as a necessary form of protection or as a violation of their autonomy

The tension between violation and protection described by patients mirrors the ethical dilemma between the fundamental bioethical principles of respect of autonomy and beneficence that is often reported in the debate about coercion. Previous studies have shown that professionals also displayed ambivalent attitudes towards the use of coercion and struggled at times to find a balance [73,74]. Indeed, when facing coercion, different values are at stake and opposed, both within the single individual and among different persons (patients, professionals, family members, etc.), making the decision-making process ethically challenging for all involved parties [75].

Hospitalisation could be perceived as a form of protection or as a violation by both voluntarily and involuntarily hospitalized patients, confirming the limitations of using formal compulsion as a measure of perceived coercion [[1], [2], [3], [4],^16^,^76^,^77^]. Patients who described the hospital experience more positively, often perceived their situation before admission as critical and dangerous for themselves or for others. Several quantitative studies had previously addressed the hypothesis of a link between patients’ level of insight and perceived coercion [[78], [79], [80], [81], [82]]. However, the results are inconclusive, with some studies showing a significant association [79,80,82], and others finding no direct relationship [78,81]. Among the qualitative studies included in this review, only one dealt directly with this question [72]. The authors concluded that even though some differences existed between patients with high and low awareness of illness in terms of negative perceptions of care, other aspects of experienced coercion were more universal and common across patients. Further research is mandatory in order to draw any conclusion on this topic.

### 4.2 Synthesized finding 2: patients perceived coercion when they lacked involvement in the decision-making process

The importance of patients’ voice, information and involvement in the decision-making process is in line with what observed in previous studies [21,83]. Patients want to have a voice and be heard despite their illness, crisis, institutional rules or struggle for power. Moreover, while the provision of information to patients was found to be associated with more positive admission experiences [84], both withheld and misleading information could lead to experienced coercion [6,85]. The amount of information received and the degree of collaboration in treatment decisions were identified as determinant factors even during voluntary admission [28].

### 4.3 Synthesized finding 3: patients perceived coercion when they experienced unsupportive, disrespectful and unreliable relationships

Collaboration is also a key ingredient of therapeutic alliance, defined as the degree of agreement between professionals and patients on therapeutic goals and tasks [86,87]. Beside collaboration, therapeutic alliance requires also the construction of a positive emotional bond based on interpersonal factors such as trust, understanding and respect [88]. The link between therapeutic alliance and experienced coercion has been proven for both voluntary and involuntary patients [89]. Our results confirmed this association, revealing that patients' experience of coercion depended more on the quality of interpersonal relationships, especially with staff, and the perceived procedural fairness of the admission process than on coercive measures themselves. In 1995, “*procedural justice*” was identified as the strongest predictor of perceived coercion by Lidz et al. [90]. This result was later strengthened by several studies showing that perceiving treatment as proportional, fair, inclusive and respectful could reduce the feeling of being coerced and enhance cooperation [4,5,[91], [92], [93], [94], [95]]. All these elements were identified in our review as being at the core of the patients' experience of coercion.

According to the procedural justice theory, being treated fairly is of crucial importance in shaping interactions, independently of their outcomes [96]. This is in line with what observed in a recent study, showing that, when taken independently, treatment effectiveness significantly affected patients’ satisfaction with care but that its effect disappeared when controlling for perceived fairness, the stronger predictor of satisfaction [97].

### 4.4 Synthesized finding 4: patients perceived coercion when they experienced hospital treatment as ineffective and unsafe

Although to a lesser extent than and often in conjunction with fairness, elements related to treatment effectiveness were however reported by patients as having an impact on their experience of coercion. Mainly, patients complained about the extensive use of medication in place of activities and therapies fostering human contact and empathy. The interplay between perceived coercion, perceived fairness, perceived effectiveness and satisfaction with care should be further studied in order to disentangle these partially overlapping concepts and better understand how they influence each other.

### 4.5 Synthesized finding 5: when they felt coerced, patients resorted to various coping strategies to deal with the situation

When restrictions are perceived as fair, respectful and implemented by professionals with whom a positive relationship based on collaboration and trust has been established, acknowledgment and agreement are possible. Otherwise, patients resort to other coping strategies, none of which effective in reducing the negative impact of the experience. Patients' verbal and behavioural opposition is often interpreted by staff as an expression of their mental disorders, to which they respond with even more coercion. The ensuing resignation can increase patients’ feelings of disempowerment and amplify their loss of self-esteem, often already severely undermined. Conforming to the system and pretending acceptance can hinder the development of a truthful therapeutic alliance, with a consequent higher risk of service disengagement. Finally, fear of rejection and social discrimination may explain the urgent need expressed by some patients to move on and leave the hospital experience behind, severing all ties with healthcare services.

### 4.6 Synthesized finding 6: when perceived as coercive, the experience of hospitalisation negatively affected several areas of patients’ identity and life

Stigmatization was frequently reported by patients because of the inpatient coercive experience. This result is in line with what observed in previous studies, showing that coercion, both perceived and formal, increased the feeling of stigma, which, in turn, led to lower self-esteem, another negative outcome outlined in our review [98,99].

A higher risk of disengagement from services may also stem from the traumatic impact that the coercive experience had on some patients, causing their condition to worsen rather than improve. This result is of utmost relevance in light of the well-known already high levels of trauma exposure of people with severe mental disorders [100,101]. Other studies have previously shown the high prevalence of re-traumatization of already traumatized patients by and within health services [102] and the treatment–related traumatic experiences impact on treatment adherence [103].

### 4.7 Synthesized finding 7: patients called for less coercive and more effective alternative interventions when in a crisis

Patients suggested the use of a variety of less coercive and more helpful alternatives when in crisis. Among these, outpatient interventions, such as Assertive Community Treatment teams [[104], [105], [106]], Home Treatment teams [[107], [108], [109]] and Crisis Intervention Teams [[110], [111], [112]], were mentioned as preferable options to hospital, allowing them to remain in their environment and closer to their relatives. Although the effectiveness of these interventions has been widely proven, their implementation is still limited in many countries, reducing patients’ accessibility [113].

### 4.8 Strengths and limitations

The main strengths of this review are the structured, systematized and replicable search strategy, the rigour of the methodology and the independent screening and critical appraisal of the included studies. Moreover, the synthesis process was informed by the discussion with the research team, including a peer-researcher. Some limitations should also be mentioned. Firstly, all synthesized findings were downgraded on dependability due to the lack of information on methodological aspects in the included studies, decreasing the level of evidence on which recommendations were developed to “moderate”. Secondly, most of the included studies were conducted in Europe and there were no studies from low-income countries. This could be due to the exclusion of the grey literature and of articles written in other languages than English, French and Italian, which could have led to miss some important study. Therefore, the generalizability of our results might be reduced.

## 5 Recommendations for clinical practice and research

### 5.1 Recommendations for practice

Based on the seven synthesized findings of the review, several recommendations for practice are suggested:

1. *Foster care ethics* (synthesized finding 1). In order to reduce the tension between protection and violation and to promote ethical decision-making, care ethics should be promoted in healthcare settings. Care ethics is an ethical approach that put interpersonal relationships and the needs of others at the core of ethical decision-making [114]. In this perspective, autonomy is conceptualized as “relational” and defined as the capacity of people to shape their own life in relationship with others [115]. Therefore, respecting autonomy does not simply mean to avoid any interference with patients' lives and leave them free to decide, but to understand their self-conceptions and view of the world, and foster their self-development [116,117]. The role of ethics in decision-making should be regularly addressed in professional trainings and ethics reflection constantly encouraged within mental healthcare teams through the development of clinical ethics support services, such as moral case deliberation and ethics consultation. Open discussions with patients on the ethical challenges underling the use of coercion should also be promoted in order to improve mutual understanding and reduce negative feelings of violation. Pros and cons of both decision and non-decision to use coercion should be discussed using motivational techniques such as decisional balance [118]. Care ethics identifies five qualities as essential to ensure ethical decision-making and respect for “relational” autonomy in the care process: attentiveness, responsibility, competence, responsiveness and solidarity [116,117,119]. Putting these virtues into practice, through the next recommendations, would not only frequently help to prevent the use of coercion but also, and perhaps to a greater extent, promote a more ethical and cooperative use of it in cases where it cannot be avoided.
2. *Promote patients' voice and shared decision-making* (synthesized finding 2). Approaches to strengthen patients' voice and shared decision-making should be supported at all levels (institutions, professionals and patients) [120]. Detailed information about the reasons, course and duration of the hospitalisation and the coercive measures eventually implemented, as well as about hospital rules and patients' rights, should be provided to all patients from the earliest stages of the admission process. Even during crisis, patients maintain the ability to listen, retain and integrate the information conveyed to them. A clearer understanding of the situation would allow them to make sense of their experience and reduce anxiety. Information should be provided throughout regular conversations with patients and the extensive provision of clear informational materials. When treatment decisions have to be taken, even during emergency situations, patients should be offered alternative solutions and be transparently informed. At the same time, patients' point of view, preferences and values must be taken into account and their expertise equally valued. Giving space to patients' voice is crucial in order to ensure cooperation, counteract the power imbalance proper to the patient-professional relationship and promote a democratic model of care. The Open Dialogue (OD) approach, fostering “polyphonic conversations” in which multiple voices can co-exist and be equally valued, should be strongly encouraged in place of the institutional monological discourse [121]. Involuntary treatment and coercion result from a lack of agreement that may remain irreducible, despite the good will of all parties. Therefore, polyphony and dialogism should be upheld as much as possible even when the decision to use coercion is taken. In these very situations, professionals should always make explicit to the patients the values and objectives underlying their decision, and be open to their reactions and feelings. Clarity, transparency and openness will foster patients' understanding and perceived fairness, and will help to restore partnership. The development of individual action plans based on shared goals, and the implementation of advance statements, such as Joint Crisis Plans [122,123] could be helpful to that end. These tools would not only improve patients' feeling of being actively involved in their care, but could also offer them the opportunity to process the coercive experience, to establish shared strategies for the prevention and possible management of future crisis, and to promote therapeutic alliance. Finally, patients' voice should also be supported and promoted through peer-support and advocacy programs, implemented within or outside the institutional settings.
3. *Enhance patients' perceived closeness, respect and fairness* (synthesized finding 3). Throughout the hospitalisation, professionals should build a positive, respectful, trustful and supportive relationship with patients. Even when patients' discernment capacity is absent in certain areas, it might be preserved in others. Professionals should be always able to identify and activate the healthiest parts of the patients and work on their resources. To strengthen the relational bond, they need to foster dialogue, promote collaboration and preserve patients' self-esteem. When verbal communication is not possible, physical closeness and practical help could be alternative paths to develop a trustful relationship. Communication skills trainings, including motivational interviewing techniques, should be provided to all stakeholders. Regular contact with professionals should also be granted. For this purpose, staff continuity should be ensured and the amount of administrative tasks reduced. Human contact rather than control should be promoted. Thus, hospital rules should be kept to a minimum and applied flexibly. Patients should be offered as much as possible the freedom to move and choose how they prefer to manage certain aspects of their daily life, such as what to wear, what time to go to bed, which activities to follow, etc. Professionals must be reliable, competent, willing to listen, interested and sincerely concerned. Greater attention should be payed to patients' dignity and respect. Patients must feel taken into account as whole human being and treated fairly, despite their disorders or the coercive measures in place. Professionals should regularly discuss these issues with all voluntarily and involuntarily admitted patients to ascertain the emergence of feelings of humiliation and de-subjectivation. Understanding the patient's subjective perception of the situation is essential in order to be certain that their personal values are taken into account and respected. These elements, combined with the implementation of shared decision-making strategies (see recommendation 2), can enhance the patients' perception of procedural justice even when coercion is enacted.
4. *Promote patients' satisfaction with treatment by enhancing hospital perceived effectiveness and safety* (synthesized finding 4). Treatments offered to patients must be adapted to their subjective needs and preferences, and their impact constantly monitored and discussed with them. Besides medication, patients should be offered alternative treatments, such as individual and group psychotherapies, occupational therapy, etc. Several daily activities should also be available to help patients to relax, divert their thoughts and feel active during their stay. Through activities, patients can also experience positive social interactions with both professionals and other patients, helping the development of relationships of trust and confidence. Ward organisation in small and quiet units should be fostered, to increase patients' feelings of peace and safety. When violence occurs within the ward, debriefing sessions should be arranged to address the impact these events may have had on the directly or indirectly involved patients.
5. *Foster true collaboration and improve negotiation skills* (synthesized finding 5). Professionals should pay more attention to the strategies adopted by patients to cope with the experience of admission and the reasons behind them. Opposition may stem from the "*righteous anger*” some patients feel when confronted with a system perceived as unfair and oppressive. In these cases, showing openness and understanding towards the reasons for their anger and using de-escalation techniques instead of coercive interventions could help to rapidly reduce aggressiveness levels and open a window for communication and negotiation. Police involvement and use of force, which may enhance patients anger and sense of oppression, should be minimized and aggressiveness management skills trainings offered to all professionals taking part in the admission process. At the same time, patients' cooperation may in some cases be dictated not by genuine acceptance, but rather by second purposes or resignation. Professionals should always encourage patients to express themselves freely, reassure them about the risk of possible retaliations and stimulate their negotiation skills in order to achieve a true therapeutic alliance.
6. *Reduce the negative consequences of experienced coercion* (synthesized finding 6). Experienced coercion should be monitored regularly for both voluntarily and involuntarily admitted patients in order to prevent its consequences and increase patients' satisfaction. Furthermore, professionals should take into consideration not only the symptoms of the disorders leading to the hospitalisation and to the eventual use of coercion, but also those generated by the experience itself, whether emotional, cognitive or behavioural. All patients having formally or informally experienced coercion should be given the chance to discuss their experience with a trusted professional. Post-coercion debriefing sessions could be helpful on this matter [124]. The promotion of a trauma-informed institutional culture, based on principals of empowerment, choice, collaboration, safety and trustworthiness could also reduce the risk of treatment-related trauma [125,126]. Finally, the development of post-discharge support programs specifically designed to help patients to process the experience of coercion, to regain a sense of empowerment and self-esteem, and to restore their confidence in potentially damaged relationships with relatives and professionals should also be encouraged.
7. *Promote the development and implementation of alternative interventions* (synthesized finding 7). Access to alternative outpatient interventions, such as Assertive Community Treatment teams [[104], [105], [106]], Home Treatment teams [[107], [108], [109]] and Crisis Intervention Teams [[110], [111], [112]], should be made available to all patients in crisis. Interventions should be proposed with due consideration of the patient's personal strategies, resources and needs, such as their activities, habits, daily rhythms, social relationships, living conditions, etc. Professionals involved in the admission process should be informed about alternative interventions available in their region and encouraged to offer them to patients. When coercion cannot be avoided, the principle of proportionality should be respected and human contact granted throughout the application of the measure (see recommendation 3). Restrictions should cease as soon as possible and patients' should be engaged in open discussion about why the measure was enacted, what impact it had on them and how they would prefer to be helped in similar situation in the future. As mentioned in recommendation 2, advanced statements, by defining beforehand which treatments are preferred by patients and which are to be avoided, can be very useful tools to guide professionals' choices when dealing with crisis and respect patients' preferences.

### 5.2 Recommendations for research

Based on the review findings, several recommendations for research are suggested:

1. *Improve the methodological quality and cultural variation of future qualitative studies.* Our review revealed that, despite the large number of qualitative studies published on this topic, especially in the past decade, several presented methodological shortcomings. Future studies should strengthen their methodological quality, especially in terms of reporting the research methodology and the influence of the researcher on the research. Moreover, since most of the included studies were from Europe, especially UK and Ireland, and there were no studies from low-income countries, further research from a greater variety of countries is needed to fill this gap.
2. *Promote research on experienced coercion involving voluntary patients.* Very few qualitative studies focused on the experience of coercion of voluntary patients. Exploring the feeling of coercion of this group of patients would be of crucial importance in order to better understand more subtle forms of coercion, improve professionals' awareness about their use, and reduce their negative effects.
3. *Clarify how determinants of experienced coercion interact with each other and with the experience outcomes.* Further research should try to disentangle the interplay between experienced coercion, perceived fairness, perceived effectiveness and satisfaction with treatment in order to develop best-targeted interventions. The role of insight should also be clarified.
4. E*xplore the psychosocial impact of experienced coercion on patients.* Further quantitative and qualitative studies should aim to a better understanding of the link between experienced coercion and patients' self-esteem, stigma and self-stigma.
5. *Develop and evaluate targeted intervention to reduce experienced coercion and its negative impact on patients.* Besides alternative interventions to coercion, future studies should aim to develop and test programs able to support patients during and after involuntary treatment, to help them process the experience of coercion and to reduce its negative impact on their future engagement with services.

## 6 Conclusions

The experience of coercion is a complex phenomenon, only partly explained by having been subjected to coercive measures. Due to its potentially detrimental impact on patients’ prognosis, satisfaction and engagement with care, the development of interventions and clinical practices able to tackle its determinants and minimise its consequences is mandatory. This review, through the first aggregative synthesis of the qualitative evidence in this field, improved the understanding of experienced coercion. Several recommendations for research and practice were suggested based on its results. For these points to be effectively implemented, policymakers must support them through their vision and policies, promoting a profound change in the organisation and culture of the mental health system. Patient involvement in the design, development and evaluation of this change, as well as in research activities, is strongly recommended.

## Author contribution statement

All authors listed have significantly contributed to the development and the writing of this article.

## Funding statement

This research did not receive any specific grant from funding agencies in the public, commercial, or not-for-profit sectors.

## Data availability statement

No data was used for the research described in the article.

## Declaration of interest's statement

The authors declare no competing interests.
