# Supplementary material for: Feeling coerced during voluntary and involuntary psychiatric hospitalisation: A review and meta-aggregation of qualitative studies
Source: Heliyon. 2023 Feb 2;9(2):e13420. doi: 10.1016/j.heliyon.2023.e13420 (PMC9937983; doi:10.1016/j.heliyon.2023.e13420)
Supplement: Multimedia component 1 [file mmc1.pdf]

Supplementary File 1

Feeling coerced during voluntary and involuntary psychiatric hospitalisation: a review and meta-aggregation of qualitative studies / Benedetta Silva, Mizue Bachelard, Joëlle Rosselet Amoussou, Debora Martinez, Charlotte Bonalumi, Charles Bonsack, Philippe Golay, Stéphane Morandi

Bibliographic database search strategies

Embase.com

101 references found, June 15, 2022

('mental disease'/exp OR 'psychiatry'/exp OR 'mental health care'/exp OR 'mental patient'/exp OR 'mental health'/exp OR ((mental NEXT/1 (disorder* OR disease* OR illness OR health OR healthcare OR patient* OR hospital*)) OR "mentally ill" OR psychiatric OR psychiatry OR (psychic NEXT/1 (disease* OR disorder*))):ab,ti,kw) AND ('involuntary commitment'/exp OR 'persuasive communication'/exp OR (coercion* OR coercive* OR "commitment of mentally ill" OR constraint* OR ((involuntary OR compulsory OR mandatory OR forced) NEAR/3 (admission* OR admitted OR care OR commitment* OR medicated OR medication OR order* OR psychiatr* OR treatment* OR hospitalisation* OR hospitalization* OR hospitali?ed OR patient*)) OR "legal commitment*" OR leverage):ab,ti,kw) AND ((perceived OR perception OR experience* OR attitude* OR view OR views OR perspective OR perspectives) NEAR/3 (coercion OR coercive* OR involuntary OR compulsory OR mandatory OR commitment)):ab,ti,kw AND (qualitative OR themes):ab,ti,kw

Ovid MEDLINE(R) ALL

91 references found, June 15, 2022

(exp mental disorders/ OR exp psychiatry/ OR exp mental health services/ OR exp mentally ill persons/ OR exp "hospitals, psychiatric"/ OR exp psychiatric nursing/ OR exp mental health/ OR ((mental ADJ1 (disorder* OR disease* OR illness OR health OR healthcare OR patient* OR hospital*)) OR "mentally ill" OR psychiatric OR psychiatry OR (psychic ADJ1 (disease* OR disorder*))).ab,ti,kf.) AND (exp commitment of mentally ill/ OR exp coercion/ OR (coercion* OR coercive* OR "commitment of mentally ill" OR constraint* OR ((involuntary OR compulsory OR mandatory OR forced) ADJ3 (admission* OR admitted OR care OR commitment* OR medicated OR medication OR order* OR psychiatr* OR treatment* OR hospitalisation* OR hospitalization* OR hospitali#ed OR patient*)) OR "legal commitment*" OR leverage).ab,ti,kf.) AND ((perceived OR perception OR experience* OR attitude* OR view OR views OR perspective OR perspectives) ADJ3 (coercion OR coercive* OR involuntary OR compulsory OR mandatory OR commitment)).ab,ti,kf. AND (qualitative OR themes).ab,ti,kf.

APA PsycINFO Ovid

94 references found, June 15, 2022

(exp mental disorders/ OR exp psychiatry/ OR exp mental health/ OR exp mental Health services/ OR exp psychiatric patient/ OR ((mental ADJ1 (disorder* OR disease* OR illness OR health OR healthcare OR patient* OR hospital*)) OR "mentally ill" OR psychiatric OR psychiatry OR (psychic ADJ1 (disease* OR disorder*))).mp.) AND (exp "commitment (psychiatric)"/ OR exp involuntary treatment/ OR exp coercion/ OR (coercion* OR coercive* OR "commitment of mentally ill" OR constraint* OR ((involuntary OR compulsory OR mandatory OR forced) ADJ3 (admission* OR admitted OR care OR commitment* OR medicated OR medication OR order* OR psychiatr* OR treatment* OR hospitalisation* OR hospitalization* OR hospitali#ed OR patient*)) OR "legal commitment*" OR leverage).mp.) AND ((perceived OR perception OR experience* OR attitude* OR view OR views OR perspective OR perspectives) ADJ3 (coercion OR coercive* OR involuntary OR compulsory OR mandatory OR commitment)).mp. AND (qualitative OR themes).mp.

Web Of Science Core Collection

120 references found, June 15, 2022

TS=((("mental" NEAR/1 (disorder* OR disease* OR "illness" OR "health" OR "healthcare" OR patient* OR hospital*)) OR "mentally ill" OR "psychiatric" OR "psychiatry" OR ("psychic" NEAR/1 (disease* OR disorder*))) AND (coercion* OR coercive* OR "commitment of mentally ill" OR constraint* OR (("involuntary" OR "compulsory" OR "mandatory" OR "forced") NEAR/3 (admission* OR "admitted" OR "care" OR commitment* OR "medicated" OR "medication" OR order* OR psychiatr* OR treatment* OR hospitalisation* OR hospitalization* OR "hospitalized" OR patient*)) OR "legal commitment*" OR "leverage") AND (("perceived" OR "perception" OR experience* OR attitude* OR "view" OR "views" OR "perspective" OR "perspectives") NEAR/3 ("coercion" OR coercive* OR "involuntary" OR "compulsory" OR "mandatory" OR "commitment")) AND ("qualitative" OR "themes"))

Cochrane Database of Systematic Reviews

4 references found, June 15, 2022

((mental NEXT/1 (disorder* OR disease* OR illness OR health OR healthcare OR patient* OR hospital*)) OR "mentally ill" OR psychiatric OR psychiatry OR (psychic NEXT/1 (disease* OR disorder*))):ab,ti,kw AND (coercion* OR coercive* OR "commitment of mentally ill" OR constraint* OR ((involuntary OR compulsory OR mandatory OR forced) NEAR/3 (admission* OR admitted OR care OR commitment* OR medicated OR medication OR order* OR psychiatr* OR treatment* OR hospitalisation* OR hospitalization* OR hospitalized OR patient*)) OR (legal NEXT commitment*) OR leverage):ab,ti,kw AND ((perceived OR perception OR experience* OR attitude* OR view OR views OR perspective OR perspectives) NEAR/3 (coercion OR coercive* OR involuntary OR compulsory OR mandatory OR commitment)):ab,ti,kw AND (qualitative OR themes):ab,ti,kw
