# Supplementary material for: Feeling coerced during voluntary and involuntary psychiatric hospitalisation: A review and meta-aggregation of qualitative studies
Source: Heliyon. 2023 Feb 2;9(2):e13420. doi: 10.1016/j.heliyon.2023.e13420 (PMC9937983; doi:10.1016/j.heliyon.2023.e13420)
Supplement: Multimedia component 2 [file mmc2.pdf]

Supplementary Table 1

Meta-aggregation results: synthesised findings and categories

Synthesised findings	References	Categories	Original findings
**1. Patients perceived the hospitalisation and its restrictions either as a necessary form of protection or as a violation of their autonomy**	20	Being violated and losing control (16)	Experiences of compulsory admission (U)<br>Medication (U)<br>Being restricted in autonomy (U)<br>Feeling out of control during hospitalisation (U)<br>Unjust infringement of autonomy (U)<br>Patients' feelings about deprivation of their liberty (U)<br>Professionals remove service user's liberty (U)<br>Regaining liberty (U)<br>Appeal to patient rights (U)<br>Excessive or unnecessary use of force (U)<br>Feeling violated (U)<br>Being confined (U)<br>Feeling trapped and coerced (U)<br>An unnecessary overreaction (U)<br>Getting help (U)<br>The need for involuntary admission (U)<br>Ambivalence on justification (U)<br>Negative emotions (U)<br>Therapy versus oppression (U)<br>The ward as a detention camp (U)<br>Lack of control about decision making in the hospital – freedom (U)<br>ITO was experienced as an intrusion into their liberty and physical integrity (U)
		Being protected and cared for (13)	Medication (U)<br>Being protected and cared for (U)<br>Mentally unwell - at risk before admission (U)<br>Need for coercive intervention - not recognising problems when unwell (U)<br>Averting risk and feeling safe in hospital (U)<br>Coercion seen as help and care (U)<br>Gaining perspective (U)<br>Coercion was necessary as protection (U)<br>A necessary emergency brake (U)<br>Getting help (U)<br>Perception of the impact of admission and diagnosis (U)<br>Ambivalence on justification (U)

			The ward as a hotel (U)<br>Benefits of involuntary hospitalisation in terms of risk reduction – safety (U)<br>The ITO protects from harm (U)
		Feeling ambivalent (4)	Ambivalent group (U)<br>Moving on? (U)<br>The need for involuntary admission (U)<br>The mixed group (U)
		Feeling unwell (4)	Mentally unwell - at risk before admission (U)<br>Deteriorating mental health of service users (U)<br>Diminishing self-mastery (U)<br>Convergence between the parties' understanding of mental disorder (U)
		Type of coercion (4)	Coercive measures (U)<br>Treatment pressures (U)<br>Informal coercion (U)<br>Restrictions on leaving the ward (U)<br>Restrictions on communication (U)<br>Confiscation of property (U)<br>Regulation and informal coercion (U)<br>Pressure to improve adherence to recommended treatment (U)<br>Pressure to improve adherence to social norms (U)<br>Explicit statements (U)<br>Things that go unsaid (U)<br>The material surroundings (U)
**2. Patients perceived coercion when they lacked involvement in the decision-making process**	20	Being informed (16)	Receiving help with understanding (U)<br>Information and advice following compulsory admission (U)<br>Being outside and not seen or heard (U)<br>Feeling out of control during hospitalisation (U)<br>Lack of information or good reason (U)<br>The early days (U)<br>Lack of informational and emotional support (U)<br>Person-centered encounters (U)<br>Not being involved in one's own care (U)<br>Being involved in one's own care (U)<br>Things that go unsaid (U)<br>A practice in need of improvement (U)<br>Information about the detention process (U)<br>Humanising care (U)<br>Dearth of knowledge and advocacy (U)<br>Captured in silence (U)

			Staff potential to impact on ITO and hospital experiences (U)<br>Provision of information about the ward rules (U)<br>Provision of information about ITO conditions (U)<br>Understanding their ITO conditions and the ward expectations (U)
		Participating in care (11)	To participate (U)<br>Inclusion (U)<br>Being respected as an individual (U)<br>Not participating sufficiently in the admission and treatment process (U)<br>Participating in the admission and treatment process (U)<br>Not being heard or no option (U)<br>Experience of treatment (U)<br>Encountering humanising care (U)<br>Not being involved in one’s own care (U)<br>Being involved in one's own care (U)<br>Lack of control about decision making in the hospital – freedom (U)<br>Relationships based on partnerships (U)<br>Having input into their treatment (U)
		Preserving autonomy (6)	To be independent (U)<br>Being protected and cared for (U)<br>Physical resistance (U)<br>Encountering humanising care (U)<br>Lack of control about decision making in the hospital – freedom (U)<br>Us and them (U)
**3. Patients perceived coercion when they experienced unsupportive, disrespectful and unreliable relationships**	18	Feeling respected and treated fairly (14)	Acknowledgment as a human being (U)<br>Beneficent motivation (U)<br>Respect (U)<br>Being violated by intrusion on physical integrity and human value (U)<br>Not feeling respected - cared for (U)<br>Feeling respected - cared for (U)<br>Experience of treatment (U)<br>Encountering humanising care (U)<br>Person-centered encounters (U)<br>Being an inferior kind of human being (U)<br>Being a human being like other people (U)<br>Nonverbal communication (U)<br>The quality of the personal relationship (U)<br>A practice in need of improvement (U)

			Human devaluation (U)<br>Considering, listening and care in personal regard – respect (U)<br>Exclusively seen as a patient (U)<br>Us and them (U)<br>De-subjectivation – Patients (U)<br>Positive encounters (U)<br>Staff potential to impact on ITO and hospital experiences (U)<br>Being able to look beyond the illness (U)
		Being in contact with staff and getting support (14)	Receiving good care (U)<br>Receiving needed shelter (U)<br>Experience of relationships and interactions (U)<br>Being outside and not seen or heard (U)<br>Being protected and cared for (U)<br>Experience of treatment (U)<br>Encountering humanising care (U)<br>Lack of informational and emotional support (U)<br>Person-centered encounters (U)<br>Receiving care perceived as meaningless and not good (U)<br>Receiving good care (U)<br>The quality of the personal relationship (U)<br>A practice in need of improvement (U)<br>The need for therapeutic care (U)<br>Relationship with the staff (U)<br>Humanising care (U)<br>De-subjectivation – Staff (U)<br>Broken contact (U)<br>Captured in silence (U)<br>Positive encounters (U)<br>Staff potential to impact on ITO and hospital experiences (U)<br>Feeling connected (U)<br>Finding time despite the busyness of the ward (U)
		Trusting the other (5)	Beneficent motivation (U)<br>Qualifications – formal and informal (U)<br>Deceit (U)<br>Trust in health personnel (U)<br>The quality of the personal relationship (U)<br>Positive encounters (U)<br>Staff potential to impact on ITO and hospital experiences (U)

		Interacting with family, friends and other patients (4)	Experience of relationships and interactions (U)<br>The quality of the personal relationship (U)<br>Relationship with families (U)<br>Being protected and cared for (U)
**4. Patients perceived coercion when they experienced hospital treatment as ineffective and unsafe**	10	Hospital perceived as effective (10)	Receiving care in a healing setting (U)<br>Hospital treatment not effective/Need for alternative treatment (U)<br>Need for coercive intervention - not recognising problems when unwell (U)<br>Managing mental health (on the psychiatric ward) (U)<br>Gaining perspective (U)<br>Receiving care perceived as meaningless and not good (U)<br>The institutional setting (U)<br>Perception of the impact of admission and diagnosis (U)<br>The necessity of treatment (U)<br>Benefits (U)<br>Having a safe space to reflect on their experience (U)
		Hospital perceived as a safe place (6)	Need for hospital treatment and safety (U)<br>Need for coercive intervention - not recognising problems when unwell (U)<br>Managing mental health (on the psychiatric ward) (U)<br>Gaining perspective (U)<br>The institutional setting (U)<br>Having a safe space to reflect on their experience (U)
**5. When they felt coerced, patients resorted to various coping strategies to deal with the situation**	8	Acknowledging and agreeing (2)	Reasons for deprivation of liberty as perceived by patients (U)<br>Agreeing and accepting (U)
		Conforming (4)	Moving on? (U)<br>Playing ball (U)<br>Emotional state (U)<br>Conforming (U)
		Resisting (3)	Fighting or resisting (U)<br>Resisting the system (U)<br>Emotional state (U)
		Resigning (3)	Resignation (U)<br>Respecting the staff (U)<br>The necessity of treatment (U)
		Moving on (2)	Moving on? (U)<br>Over, not to be recalled (U)
**6. When perceived as coercive, the experience of hospitalisation negatively affected several areas of patients' identity and life**	8	Well-being and mental health (4)	Recovery in the community (U)<br>Living with the consequences of involuntary hospitalisation (U)

			Admission-induced trauma (U)<br>Impact on health (U)<br>Positive changes (U)
		Relationships and social life (6)	Experience of relationships and interactions (U)<br>Unjust infringement of autonomy (U)<br>Recovery in the community (U)<br>Living with the consequences of involuntary hospitalisation (U)<br>Preserving sense of self (U)<br>Impact on relationships and community life (U)<br>Motivation for political engagement (U)<br>Signing the application and the perceived impact on relationship (U)
		View of self (5)	Views of self (U)<br>Living with the consequences of involuntary hospitalisation (U)<br>Admission-induced trauma (U)<br>Impact on self-esteem and sense of self (U)<br>Emotional state (U)
		Activities and daily life (2)	Unjust infringement of autonomy (U)<br>Impact on relationships and community life (U)
**7. Patients called for less coercive and more effective alternative interventions when in a crisis**	5	Outpatient services and mobile teams (5)	Need for non-coercive treatment (U)<br>Managing mental health (U)<br>Outpatient care (U)<br>A practice in need of improvement (U)<br>Interventions and alternative suggestions (U)
		Human contact with professionals and other patients (3)	Human contact and handling problems better (U)<br>A practice in need of improvement (U)<br>Interventions and alternative suggestions (U)
		Voluntary admission and shorter coercive measures (3)	Need for non-coercive treatment (U)<br>Preventing coercion lasting too long (U)<br>Voluntary admittance (U)<br>Interventions and alternative suggestions (U)
		Personal strategies (2)	Need for non-coercive treatment (U)<br>Managing mental health (U)

Note: U=unequivocal; E=equivocal; UN=unsupported.
